# Harnessing the potential of practice‐based clinical optometry research in the United Kingdom

**DOI:** 10.1111/opo.13079

**Published:** 2022-12-13

**Authors:** Laura J. Taylor, Angharad Hobby, Michael Bowen, Jasleen K. Jolly, Robert E. MacLaren

**Affiliations:** ^1^ Nuffield Laboratory of Ophthalmology, Nuffield Department of Clinical Neurosciences University of Oxford Oxford UK; ^2^ Oxford Eye Hospital Oxford University Hospitals NHS Foundation Trust Oxford UK; ^3^ Cwm Taf Morgannwg University Health Board Wales UK; ^4^ The College of Optometrists London UK; ^5^ Vision and Eye Research Institute Anglia Ruskin University Cambridge UK

**Keywords:** healthcare, optometry research, practice‐based evidence, practice‐based research

## Abstract

Research is the core of evidence‐based practice across all healthcare, in order to ensure optimum patient care. The College of Optometrists is a national standard setting institution for optometric practice in the United Kingdom. However, the standards are only as good as the available evidence, and currently there is little evidence relating directly to optometric practice. The National Institute of Health and Care Research, the General Medical Council and The College of Optometrists, amongst others, have published research strategies describing ambitious plans to expand the scope of healthcare research. The aim of this article is to raise awareness of these government initiatives and consider how they may relate to optometric practice. To improve optometrist research engagement, we need to address the barriers to research and implement strategies to overcome them. There are many opportunities to support research, with different degrees of involvement, from signposting patients to research studies, supporting recruitment or collecting data for a multicentre clinical trial, as well as undertaking an individual research project. Healthcare research is changing and there is scope for more practice‐based research activities in optometry. Research should not be a solo endeavour but a multi‐disciplinary effort. Greater collaborations across all stakeholders, including primary care, secondary care, academia, regulators and industry is needed to make this possible.

**Key points**

- In the United Kingdom, regulatory authorities are keen to increase the scope and capacity of healthcare research, to build evidence‐based practice and improve patient care.
- In optometry, the barriers to research engagement need to be addressed, for both patients and clinicians, and strategies implemented to overcome them.
- There are lots of opportunities for optometrists to engage with research both in practice and beyond, with different levels of involvement, but greater collaborations across eyecare stakeholders and research funders are required.

## PURPOSE

Research is vital for evidence‐based practice across all healthcare settings, including optometry, to ensure the best patient care. The College of Optometrists is a national standard setting institution for optometric practice in the UK. It seeks to provide evidence‐based standards of practice. However, there is limited evidence relating directly to some aspects of optometric practice to base these standards on. Furthermore, research opportunities relating directly to optometric practice are limited.

In the UK, the traditional route into optometric research is to undertake a Ph.D., often at one of the universities teaching optometry and vision science. From here, some doctorate graduates may choose to stay in academia, undertaking a post‐doctoral or lecturing position and then possibly developing their own research group. However, most optometrists elect to return to work in clinical practice, while some embark on a career in industry. For those that take these routes or who have not completed a Ph.D., what opportunities are there to be involved in research? What opportunities are there to remain research active in both primary and secondary National Health Service (NHS) eyecare? This article summarises initiatives around the future of healthcare research and discusses opportunities for research in optometric practice.

## NEW RESEARCH POLICIES AND STRATEGIES

The Department of Health & Social Care has set out bold ambitions for the future of clinical research within the UK. The latest policy document, ‘The Future of United Kingdom Clinical Research Delivery: 2021 to 2022 implementation plan’ details these ambitions. They include encouraging more clinical research across all healthcare settings, and across the entire nation by embedding research into routine NHS care.^1^

The General Medical Council has set out plans to “normalise research” so that it becomes part of routine patient care in medical practice.^2^ They have stated their commitment to ensuring continued opportunities for doctors to get involved in research at any stage of their career. The General Medical Council propose to raise the profile of research, support a research active workforce with those in research roles having protected and salaried research time, and research resources being built into healthcare budgets. There are plans to encourage those who are not actively engaged in research to be “research aware*”*, so that patients can be directed to relevant research opportunities.^2^

The National Institute for Health and Care Research (NIHR) supports research in the NHS. Although centred on England, the NIHR works closely with devolved administrations in Scotland, Wales and Northern Ireland. In 2022, the NIHR published their “areas of strategic focus” in ‘Best Research for Best Health: The Next Chapter’.^3^ These include; building capacity in public health and social care research, expanding support for clinical and applied research in underserved regions and strengthening research delivery careers (particularly in underrepresented specialisms) by supporting academic career pathways. Both primary and secondary care based optometric research could arguably fall within all these areas. Optometrists have the potential to be involved in a whole range of healthcare research and are well placed to recruit all groups of patients. Community eyecare practices are typically more accessible for patients than research centres in large cities.

The NIHR Clinical Research Network (CRN) is made up of thirty speciality groups, including ophthalmology. The CRN provides the practical support to enable high‐quality research to take place within the NHS and wider health and social care environments, including set‐up and delivery support via the study support service. The NIHR CRN with ongoing support from charities (Macular Society, Fight for Sight, Moorfields Eye Charity and Retina UK) have launched the UK Clinical Eye Research Strategy to prioritise research to help meet the high clinical demands on ophthalmology outpatient departments.^4^

The College of Optometrists is working closely with the NIHR's Ophthalmology Specialty Group to identify and develop opportunities for optometrists to participate in research projects, as well as contribute to the wider CRN infrastructure. In 2020, The College of Optometrists celebrated 40 years of research and in 2021 published their five‐year research plan.^5^ Evidence‐based health care is the foundation of clinical and professional practice, research is a vital element to guide professional standards of practice and is highly valued by The College of Optometrists. Since 2007, the college has proactively invested in promoting and developing opportunities for optometrists to gain skills and experience of practice‐based clinical research (including both primary and secondary care). Initially, through their innovation in Practice‐based Research in Optometry (iPRO) scheme (2007–2015). Then via their practice‐based research advice service and Small Grants Scheme, which provides funding for practice‐based optometrists to carry out research projects in clinical settings. The college also offers funding for Clinical Research Fellowships, though these are currently only accessible to those in secondary care roles. The college's 2021 5‐year plan aims to build further research capacity by focusing on delivering evidence in priority areas, increasing research impact, translating research into practice and building more research opportunities. Furthermore, the college aims to support the optometry profession to achieve the aspirational objectives set out by the Alliance for Useful Evidence in their 2019 report: ‘Bodies of Evidence’. The report describes how professional organisations can “champion the use of research” to ensure “evidence‐informed practice”.^6^

In Wales, reformation of the General Ophthalmic Service (GOS) contract is underway.^7^ In light of this the Welsh Optometric Committee hosted a Research Symposium. The aim of this event was to showcase current research and discuss future facilitation of practice level research in primary care optometry. During the meeting, the development of a research strategy was proposed to incorporate eyecare research involvement into future optometric practice alongside approaching changes to the GOS contract. The proposition of a research strategy is supported by the Welsh Government and follows on from similar requests in other healthcare disciplines, such as Pharmacy. Optometry Wales are working to develop this research strategy with input from various representatives (e.g., optometrists, health boards, universities, funding providers and patients). It is hoped that the research strategy can develop a strong research culture in a well‐supported optometric workforce. This will enable research to be amplified and extended, encouraging more participation in primary care to enable evaluation of services within and beyond the new contract for both patient and practitioner benefit.

In research‐active healthcare settings, patients report better experiences and show improved healthcare outcomes.^8^ Following the pandemic, there is a strong motivation to grow the scope and breadth of research across healthcare in the UK to improve patient care. This includes getting more healthcare professionals, both in primary and secondary care, involved in research. Despite these ambitious strategies, it is unclear how optometry across the UK is going to achieve making healthcare research a part of routine ophthalmic care.

## CHALLENGES

Research in primary and secondary care settings has untapped potential but deciphering how this becomes a reality is challenging. Existing barriers to research must be addressed and strategies provided to overcome them. Many of these barriers are not unique to optometry. Table 1 summaries some of these barriers, although this is not an exhaustive list.

**TABLE 1:** A summary of the barriers to practice‐based research in optometry

Barriers to practice‐based research
Impact on daily practice
Research set‐up processes are complex and time consuming
Research co‐ordination and recruitment are time consuming
Many practices/practitioners are already over burdened
Financial
Optometry is neither medical nor allied health; this excludes optometry from some research grants
Insufficient funding to enable research (e.g., financial support to scale back clinical involvement, or employ research dedicated staff)
Lack of payment structures to compensate primary care practices for research activities
Skillset
Research skills knowledge gap (e.g., Good Clinical Practice training)
Lack of research experience amongst workforce, including optometrists who have completed doctoral awards
Many academic optometrists focus on vision sciences as opposed to practice‐based research
Lack of a clear linkage between developing research skills or having research activities on a professional curriculum vitae and career progression.
Governance & Contracts
Limited research governance and contractual support outside established research institutions
Consistency in data collection, different testing equipment and computer systems may limit data acquisition and transfer

The General Medical Council acknowledge that clinicians should have protected time in their job to carry out professional activities such as research, but often this is deprioritised to manage increasing workloads and patients. They suggest that clinical activities should be structured to facilitate research delivery and have access to infrastructure to support research engagement. They emphasise that clinicians should be empowered to recognise the need for research in their professional role.

Discussion of the barriers and challenges faced in practice based clinical research and how we can overcome these warrants a full workup and could form the basis of a comprehensive clinical optometric research strategy. Expanding practice‐based research should not be a solo endeavour. To ensure high quality and effective research activities, strong collaborations between all nations and eyecare stakeholders are required.

## CURRENT RESEARCH OPPORTUNITIES

**FIGURE 1:**
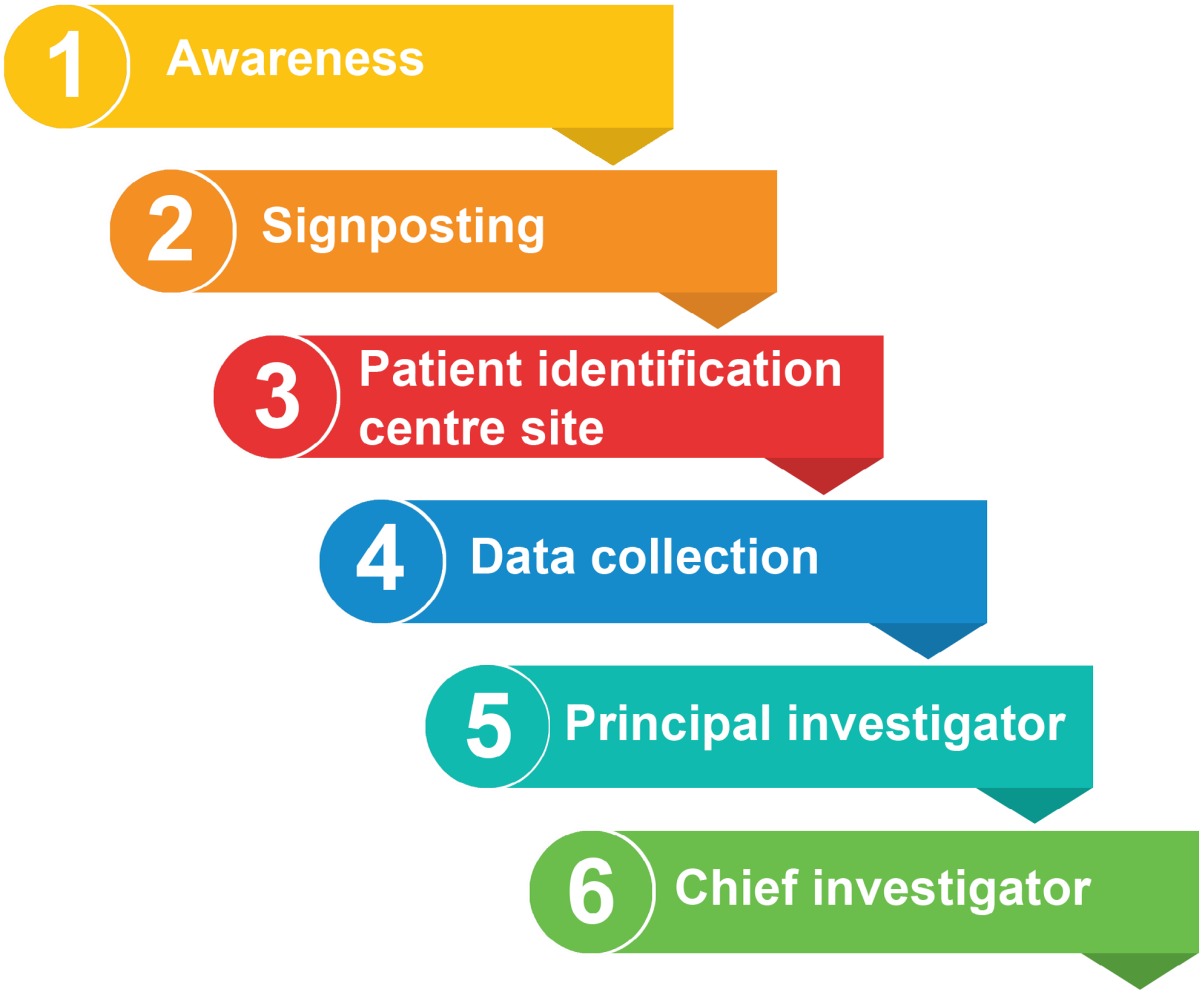
Details the levels of practice‐based research involvement and activity

To expand optometric healthcare research, building on current research opportunities is a logical place to start. Figure 1 summaries the levels of research activity. There are numerous opportunities to get involved in optometric research, but increased awareness is needed to encourage greater engagement.

There is a perception that individuals require a Ph.D. to be involved in research. While undertaking a Ph.D. provides useful research skills, it is not a prerequisite for involvement in research activities. The General Medical Council suggested that you can be research aware and support research without being actively involved. In England, the Local Optical Committee Support Unit keep an updated list of research studies for optometrists to either take part in or signpost patients to.^9^ If optometrists had greater awareness of research studies taking place in their area, they could signpost patients accordingly or put up study advertisements in the waiting room. Local Optical Committees could have appointed research leads to promote engagement. Providing patients with information about research studies is arguably part of our duty of care since patients can benefit from being involved in research.

The next level up could involve a practice becoming a ‘Patient Identification Centre (PIC)’ site. This includes screening patients lists to identify those eligible for a specific trial. Under the study sponsors' guidance, these patients are contacted and recruited to participate in the research. An example of this approach includes identifying a cohort of patients with a specific eye condition, e.g., early age‐related macular degeneration or a particular contact lens. The PIC site is then reimbursed for each participant that is enrolled in the study.

Research participation does not need to involve setting up and co‐ordinating an entire study or writing a research paper or thesis. It could include supporting data collection for commercially sponsored trials such as the ‘TELESCOPE’ study, sponsored by Gyroscope Therapeutics.^10^ This involves collecting saliva samples from patients with macular degeneration and sending them for genetic testing. The aim is to identify those patients with rare genetic variants that may be associated with the macular disease. These patients are invited to take part in other research studies, and the optometric practice is reimbursed following successful recruitment.

The increasing number of shared care services makes now an important time to embrace practice‐based research to ensure that we are providing the best and most up‐to‐date patient care. The ‘Fenetre Study: quality‐assured follow‐up of quiescent neovascular age‐related macular degeneration by non‐medical practitioners’ is a prime example of shared‐care optometry research opportunities. The study involves 70 optometric practices and 16 hospital sites. It aims to assess the safety and quality of community led follow‐up of patients with neovascular age‐related macular degeneration and to compare with hospital based follow up.^11^

In ophthalmic clinical trials, optometrists are often relied upon to collect clinical data. However, there are opportunities for optometric input to benefit clinical trial design, optimise visual function testing methodology and identify the most appropriate visual function endpoints. The Oxford Biomedical Research Centre (supported by the NIHR) part‐funds the Oxford retinal gene therapy programme. This employs optometrists to work specifically on improving outcome measures used in the ophthalmic clinical trials, exemplifying the importance of incorporating optometric skills within ophthalmic research.

The popularity of level seven postgraduate courses in optometry, including independent prescribing, suggests a high level of motivation in the optometric workforce to develop knowledge and scope of practice. However, many professional certificates and diplomas do not include research opportunities, unless masters or doctoral awards are undertaken. Is this a missed opportunity for research engagement? Many universities now offer Ph.D. by publication doctoral awards, which have proven popular with other healthcare professionals in practice and could work well in optometry. These research experiences could provide the foundation for more research opportunities and develop practice‐based research activities. Alternatively, a programme like the American Academy of Optometry Clinical Investigator Certification^12^ would support optometrists to lead more clinical trials. The College of Optometrists is currently working with partners at Ulster University to develop e‐learning modules designed to enable optometrists working in clinical settings to develop research skills. These modules will become available in the NIHR's e‐learning platform in 2023 and 2024. Perhaps in time, these will support the emergence of specialist research optometrist or clinical academic optometrist roles that follow an established career pathway.

## CONCLUSION

It is an exciting time for healthcare research and there is scope for greater practice‐based research in optometry. Being involved in research can bring many benefits to patients as well as individual clinicians and the profession overall. However, there are barriers to overcome to enable research to become a greater part of routine eyecare. A greater research emphasis during undergraduate training and increased collaborations between primary care, secondary care, academia and industry, are necessary to nurture these practice‐based research opportunities.

## AUTHOR CONTRIBUTIONS

**Angharad Hobby:** Conceptualization (supporting); formal analysis (supporting); investigation (supporting); validation (supporting); visualization (supporting); writing – original draft (supporting); writing – review and editing (supporting). **Michael Bowen:** Conceptualization (supporting); formal analysis (supporting); validation (supporting); writing – original draft (supporting); writing – review and editing (supporting). **Jasleen K. Jolly:** Validation (supporting); writing – original draft (supporting); writing – review and editing (supporting). **Robert E. MacLaren:** Resources (supporting); supervision (lead); validation (supporting); writing – original draft (supporting); writing – review and editing (supporting).

## CONFLICT OF INTEREST

The authors report no conflicts of interest and have no proprietary interest in any of the materials mentioned in this article.
